# High extinction ratio super pixel for long wavelength infrared polarization imaging detection based on plasmonic microcavity quantum well infrared photodetectors

**DOI:** 10.1038/s41598-018-33432-9

**Published:** 2018-10-10

**Authors:** Yu Wei Zhou, Zhi Feng Li, Jing Zhou, Ning Li, Xiao Hao Zhou, Ping Ping Chen, Yuan Liao Zheng, Xiao Shuang Chen, Wei Lu

**Affiliations:** 1 State Key Laboratory of Infrared Physics, Shanghai Institute of Technical Physics, Chinese Academy of Sciences, Shanghai, 200083 China; 2 University of Chinese Academy of Sciences, Beijing, 100049 China

## Abstract

Polarization imaging detection has its unique advantage in discriminating the man-made objects from natural objects. Grating integrated super pixel for polarization imaging detection can simultaneously obtain the first three elements of the Stokes vector, which is the trend of infrared polarization imaging detection in recent years. Here, we demonstrate the first super pixel for long wavelength infrared polarization imaging detection with the extinction ratio of its four polarization directions more than 100. The measured highest polarization extinction ratio is as high as 136, which is the highest reported value of long wavelength infrared polarization imaging detection super pixel. The mechanism is attributed to the excellent mode selectivity of plasmonic microcavity according to the results of three-dimensional theoretical simulation. The experimental responses of the super pixel with four polarization directions are in good agreement with the Malus’ Law. In addition, the super pixel can accurately resolve the Stokes parameters at the same time. It is expected to develop the super pixel into a new generation of practical high-polarization-discriminating long wavelength infrared focal plane array.

## Introduction

Besides the intensity and frequency of light, polarization provides another important information for remote sensing imaging^1^. Polarization detection is the frontier and trend of many fields such as astronomy^2,3^ and bionics, of which the role is irreplaceable. It has unique advantages of identifying artificial objects such as metals and glass, and could achieve high precision without accurate calibration of radiation. Usually, we use the Stokes vector^4^ to describe the polarization state of light, which is defined as follows:

1 $${\rm{S}}=[\begin{array}{c}{\rm{I}}\\ {\rm{Q}}\\ {\rm{U}}\\ {\rm{V}}\end{array}]=[\begin{array}{c}{{\rm{I}}}_{0}^\circ +{{\rm{I}}}_{\mathrm{90}}^\circ \\ {{\rm{I}}}_{0}^\circ -{{\rm{I}}}_{\mathrm{90}}^\circ \\ {{\rm{I}}}_{\mathrm{45}}^\circ -{{\rm{I}}}_{\mathrm{135}}^\circ \\ {{\rm{I}}}_{\mathrm{RCP}}-{{\rm{I}}}_{{\rm{LCP}}}\end{array}]$$

where I is the intensity of the incident light, Q and U are related to linear polarization with four directions, V describes the circular polarization. These parameters are often normalized to the value of I so that they are all between −1 and +1. Many studies have shown that the polarization degree of natural object is very small, and generally the circular polarization component could be ignored^5^ (that is, V = 0).

The degree of linear polarization (DoLP), which indicates the performance of polarization, is defined as

2 $${\rm{DoLP}}=\frac{\sqrt{{Q}^{2}+{U}^{2}}}{I}(0\,\le \,DoLP\,\le \,1)$$

Another index to directly describe the polarization performance is the polarization extinction ratio^6^ (ER), which is defined as

3 $${\rm{ER}}({\rm{\lambda }})=\frac{{{\rm{R}}}_{{\rm{\max }}}(\lambda )}{{{\rm{R}}}_{{\rm{\min }}}(\lambda )}$$

where R_max_(*λ*) and R_min_(*λ*) are the maximum and minimum response at the wavelength of *λ*, respectively. DoLP = 0 corresponds to the condition where ER = 1, and DoLP = 1 corresponds to the condition where ER is infinitely large.

Target detection and recognition are always disturbed by bad weather, complex background and camouflage technology. The camouflage technology could effectively weaken the infrared characteristics and make it difficult for the infrared intensity detection to identify the targets. However, the DoLP of camouflaged targets changes little, which requires to obtain the first three elements of Stokes vector. The camouflaged targets would be easily detected in the polarimetric image. Thus, polarization imaging detection is of great significance for rapid identification of camouflage targets and high accuracy of alarm rate^7^. Polarization imaging detection in different wavebands is becoming one of the hotspots in recent years^8–10^.

There are several different approaches for polarization imaging detection^11^ such as rotating element, division of amplitude, division of focal plane and so on. The approach of rotating element is the easiest way to implement, but it is not suitable for rapid identification of dynamic targets. Although the approach of division of amplitude could achieve simultaneous detection, it needs multiple high flexible focal plane arrays and a large system size. In consideration of these factors, we adopt the approach of division of focal plane in this research. This kind of detector is able to get the first three elements of Stokes vector simultaneously and achieve fast processing and imaging with a single focal plane array (FPA).

It is reported that the infrared polarization detection is realized by combining the micro-polarizer array with the HgCdTe focal plane^12^, and the maximum polarization ER of the four polarization directions is 9.5. But this method is difficult especially the fabrication of the micro-polarizer array^13^ and the combination with the infrared focal plane. In the past thirty years, quantum well infrared photodetectors (QWIPs) have been developed rapidly with the III-V semiconductor technology becoming more and more mature. The studies on focal plane of polarization imaging detection based on the long wavelength infrared (LWIR) quantum well emerge endlessly^14–16^, but the ER of super pixel is generally poor. Monolithic integrated LWIR quantum well polarization imaging detection FPA has been developed (peak wavelength at 8.4 μm), but the maximum polarization ER of the four polarization directions is less than 3.

Plasmonic microcavity (PMC) could manipulate photons at subwavelength scale^17–19^ to increase the photoelectric coupling and promote the absorption of quantum well, so as to improve the quantum efficiency of QWIPs. For large mesas, previous study^20^ has shown that the metal-dielectric-metal structure (MIM) with one-dimensional (1D) gratings has a strong response enhancement and high polarization ER, but the pixel size should be small enough to achieve a large scale of FPA. On condition of pixel-level mesa, the number of gratings reduces and the size of the gratings is limited, so the situation is quite different with that of large mesas.

In this work, we take advantage of the plasmonic microcavity, and apply it to pixel-level QWIP, of which the process is totally compatible with that of FPA. We have developed the super pixel for LWIR polarization imaging detection with the ER of the four polarization directions more than 100 (DoLP > 0.980). The highest polarization ER measured with Fourier transform infrared spectrometer (FTIR) in experiment is 136 (DoLP = 0.985), which is the highest reported value of LWIR polarization imaging detection super pixel. The essence of the high ER is attributed to the double selection. On one hand, the plasmonic microcavity with 1D gratings shows an excellent mode selectivity according to the results of three-dimensional (3D) theoretical simulation. On the other hand, since quantum well could not absorb the normally incident light, the QWIPs only absorb the light selected by the plasmonic microcavity. It is this mechanism that makes the high ER possible. The experimental responses of the super pixel with four polarization directions are in excellent agreement with the Malus’ Law. We can accurately resolve the first three elements of the Stokes vector simultaneously at the pixel level. The results of this work are expected to directly develop the super pixel into a new generation of practical high polarization discriminating LWIR-FPA. High-quality polarimetric image provided by this kind of super pixel, combining with some algorithms of polarimetric image processing^21^, could yield a broad and promising future for polarization detection FPAs based on plasmonic microcavity quantum well infrared photodetectors.

## Result

### The structure of PMC-QWIP super pixel

**Figure 1:**
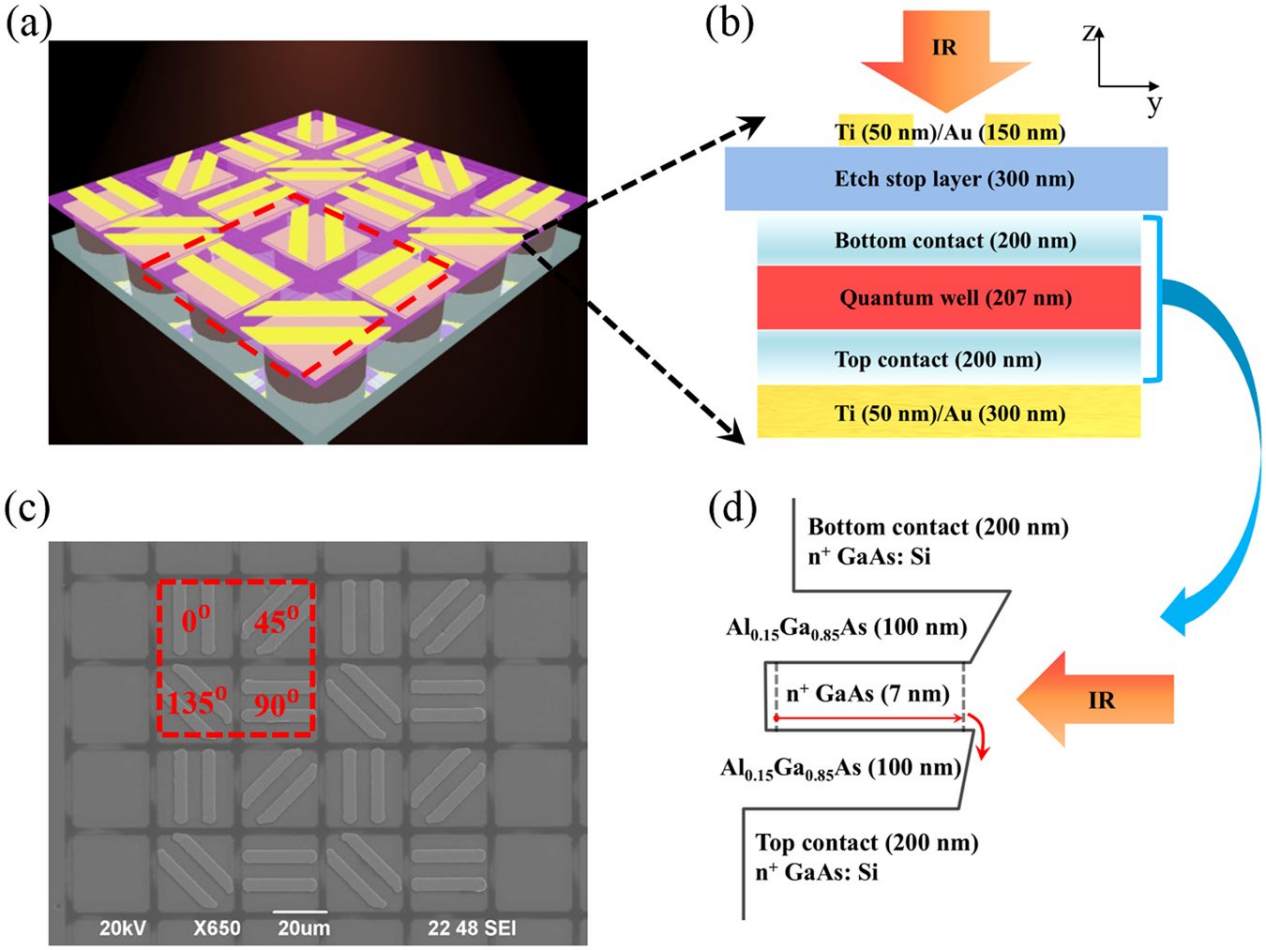
Schematic view of the super pixel of PMC-QWIP. (**a**) 3D schematic diagram of the super pixel arrays with fanout circuit. A super pixel is in the red dashed line frame. (**b**) Cross-sectional view of a PMC-QWIP pixel. (**c**) SEM image of super pixel with the angle definition of gratings. (**d**) Band diagram of the single quantum well and the thickness of sub-layers.

As shown in Fig. 1a, the PMC-QWIP super pixel arrays are bonding with the fanout circuit using indium bumps. Four subpixels with different orientations of gratings are defined as a super pixel, as shown in the red dashed line frame, and each kind of subpixel could be regarded as a channel which is able to detect the signal of linear polarization in a certain direction. The cross-sectional view of a PMC-QWIP pixel is shown in Fig. 1b. The material from bottom to top is composed of a 200 nm n-doped GaAs top contact layer (doping with Si: n = 2 × 10^17^ cm^−3^), a 207 nm single quantum well layer (100 nm Al_0.15_Ga_0.85_As/7 nm n-doped GaAs/100 nm Al_0.15_Ga_0.85_As), a 200 nm n-doped GaAs bottom contact layer and a 300 nm etch stop layer. The material is sandwiched between two layers of metal. The gratings near the etch stop layer are 200 nm thick, and the metal reflection layer is 350 nm. The size of a single pixel is 27 × 27 μm^2^, which is the common pixel size of FPA, and the center distance is 30 μm. The distance between the upper metal gratings and lower metal reflection layer is 907 nm, so the plasmonic microcavity (PMC) structure is formed, in which the incident light field is localized.

For linear polarized incident light, the PMC structure and the absorption principle of quantum well^22,23^ together determine that this kind of metal/dielectric/metal (MIM) structure with one-dimensional (1D) gratings could only couple and absorb the incident transverse magnetic (TM) polarized light, which is the basis of our high polarization discriminating device. The 0 degree gratings (G0) defined in this paper are vertical gratings, as shown in Fig. 1c, and 45 degree gratings (G45), 90 degree gratings (G90) and 135 degree gratings (G135) are defined by clockwise rotation.

### Photocurrent spectra and extinction ratio of super pixel

**Figure 2:**
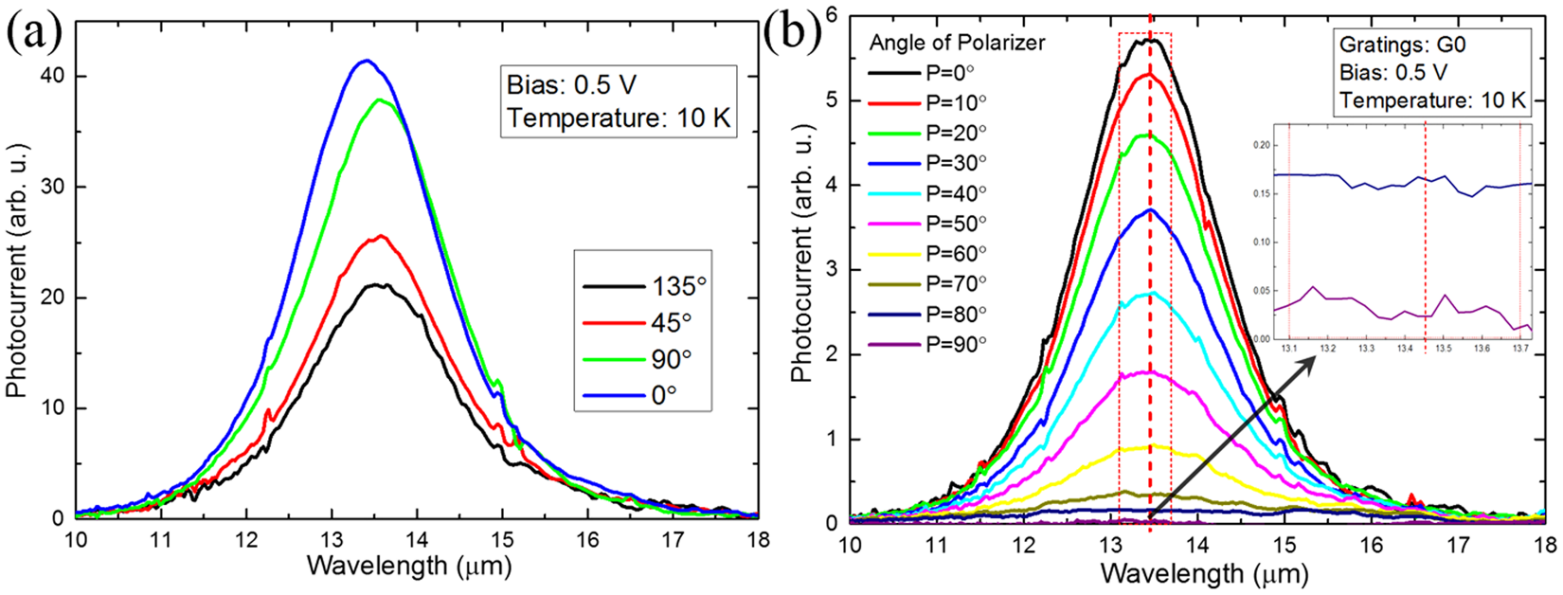
Photocurrent spectra of the super pixel and the maximum polarization extinction ratio measured. (**a**) Photocurrent spectra of the super pixel with four orientation of gratings in the same experimental condition. (**b**) Photocurrent spectra vs. wavelength (G0) at different polarization angles. The inset shows the photocurrent spectrum at great extinction. Polarization extinction ratio is calculated by the average data in the red rectangular dashed frame.

In this work, the parameters of PMC have been formerly optimized and the enhancement of responsivity mainly results from the good coupling of localized surface plasmon (LSP) mode in the microcavity. The intrinsic absorption peak wavelength of material is around 13.2 μm. The width of gratings is 5.10 μm, which leads to a responsivity enhancement peak around 13.7 μm. The photocurrent spectra are measured by Fourier transform infrared spectrometer (FTIR), as shown in Fig. 2a. The four pixels in the super pixel is measured under the same conditions. The response of G0 and G90 is larger than that of G45 and G135, which attributes to the smaller effective PMC length of G45 and G135.

Ideally, only the TM polarized incident light (the electric field component is along y direction in Fig. 1b) contributes to the photo-responsivity. The response of each polarization detection pixel with the angle of the incident polarized light ideally follows the Malus’ Law (cosine-squared rule), that is:

4 $${\rm{R}}({\rm{\theta }})={{\rm{R}}}_{0}\cdot {\cos }^{2}({\rm{\theta }}+{{\rm{\theta }}}_{0})$$

where R(θ) is the response of the pixel, R_0_ is the peak of response, θ is the angle of the incident polarized light, and θ_0_ is the initial phase. In this condition, the minimum value of R(θ) is 0 and the extinction ratio (ER) is infinitely large. However, due to some factors such as a small amount of TE polarized light coupling into the microcavity from the edge of gratings and the imperfection of incident light in polarization (ER is not infinitely large), the practical measured response of the device obey the form as follows:

5 $${\rm{R}}^{\prime} ({\rm{\theta }})={{\rm{R}}}_{0}^{^{\prime} }\cdot {\cos }^{2}({\rm{\theta }}+{{\rm{\theta }}}_{0})+{{\rm{R}}}_{1}$$

where R′(θ) is the measured response of the pixel, ${{\rm{R}}}_{0}^{^{\prime} }$ is the peak response relevant to polarization, R_1_ is the minimum value of measured response which is irrelevant to polarization. In this condition, the peak value of measured response is ${{\rm{R}}}_{0}^{^{\prime} }+{{\rm{R}}}_{1}$, and the extinction ratio is expressed by:

6 $${\rm{ER}}=\frac{{{\rm{R}}}_{0}^{^{\prime} }+{{\rm{R}}}_{1}}{{{\rm{R}}}_{1}}$$

The practical measured extinction ratio is a finite value, and a larger extinction ratio of the device means a better performance in the polarization detection. When the ER is large, R_1_ is too small to accurately measured, which is usually disturbed by thermal noise. In order to get the accurate result of ER, we fix the temperature at 10 K in experiment. The photocurrent spectra of G0 with different polarization angles are shown in Fig. 2b, and the experimental extinction ratio of the super pixel is shown in Table 1.

**Table 1:** The experimental extinction ratio of the super pixel of PMC-QWIP.

Grating Orientation	Grating Symbol	Measured Extinction Ratio
0°	G0	136
90°	G90	131
45°	G45	116
135°	G135	122

As shown in the red rectangle dashed frame in Fig. 2b, the values of spectral response change little between 13.1 μm and 13.7 μm and the peaks of spectral response are included in this range. We use the average value over the spectral range from 13.1 to 13.7 μm as the peak response to calculate the ER, which both corresponds to the ER of low spectral resolution and reduces the interference of noise. In this way, the ER of G0 pixel is 136, which is the highest reported extinction ratio of long wavelength infrared polarization detection pixel. From the data in Table 1, we can see that the measured ERs of the four subpixels of super pixel are all above 100 (DoLP > 0.980). It is suggested that the extinction ratio can be increased by more than an order of magnitude by means of PMC-QWIP super pixel in polarization detection.

### Polarization characteristics of super pixel and resolution of Stokes parameters

**Figure 3:**
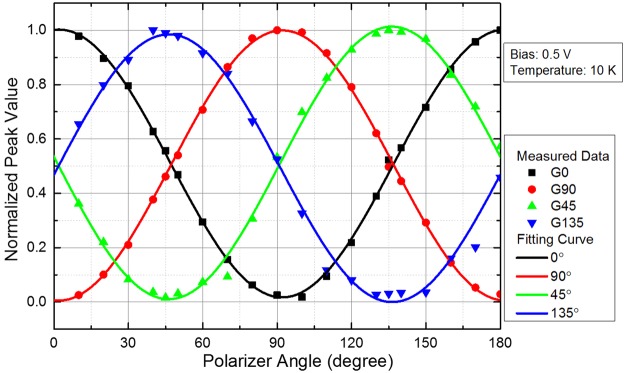
Polarization characteristics of the super pixel of PMC-QWIP. Dots are normalized measured average peak values of photocurrent, and lines are the fitting curves.

Figure 3 shows the variation in the spectral response peak of the four polarization directions of the super pixel with the angle of polarized incident light. In Fig. 3, dots are the normalized measured photocurrent peak values and lines are the fitting curves by the least square method based on the dots. The function form of the fitting is as follows:

7 $${\rm{y}}={\rm{A}}\cdot {\cos }^{2}({\rm{x}}+{\rm{B}})+{\rm{C}}$$

This function form corresponds to Equation (5) where y is the normalized photocurrent peak value corresponding to the normalized R′(θ), x is the polarizer angle which is the angle of polarized incident light, A and C correspond to normalized ${{\rm{R}}}_{0}^{^{\prime} }$, B corresponds to θ_0_. As shown in Fig. 3, the variation in the response peak of the super pixel fits well with the Malus’ Law. The PMC-QWIP super pixel has excellent performance in polarization detection.

**Figure 4:**
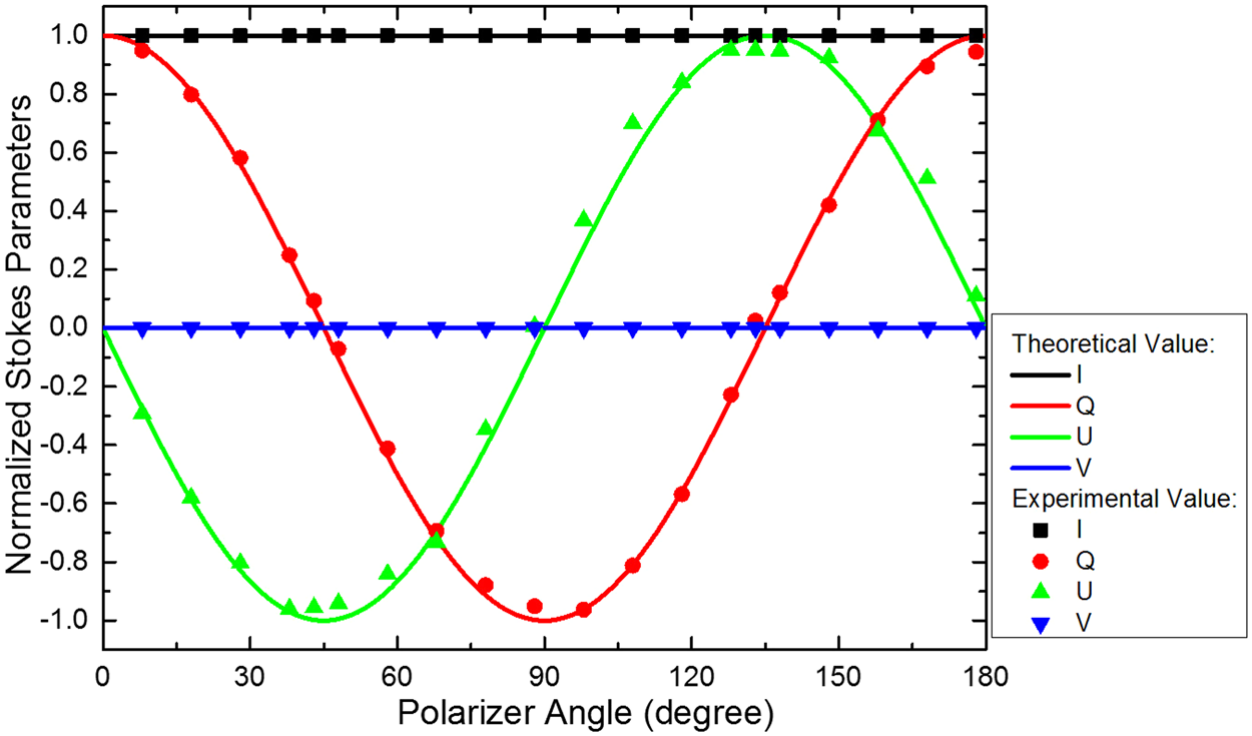
Resolution of Stokes parameters. Dots are Stokes parameters resolved by the super pixel of PMC-QWIP, and lines are the theoretical Stokes parameters.

One of the most important goals in polarization detection is to resolve the Stokes vector. Equation (1) shows that the first three elements of the Stokes vector are related to linear polarization. It indicates that we can simultaneously get the first three Stokes parameters by this PMC-QWIP super pixel. We also use the polarizer to generate linear polarized incident light and try to resolve the Stokes parameters. The resolution results of Stokes parameters by our PMC-QWIP super pixel are shown in Fig. 4, in which dots are the Stokes parameters resolved by the super pixel and lines are the theoretical Stokes parameters.

We can see from the Fig. 4 that the Stokes parameters can be well resolved by the PMC-QWIP super pixel. The excellent performance of PMC-QWIP super pixel in polarization detection is the guarantee of the resolution of Stokes vector. FPAs by using this kind of high polarization discriminating super pixel with four channels have a promising prospect in the application of polarization detection.

## Discussion

### Numerical simulation of PMC-QWIP super pixel and analysis

**Figure 5:**
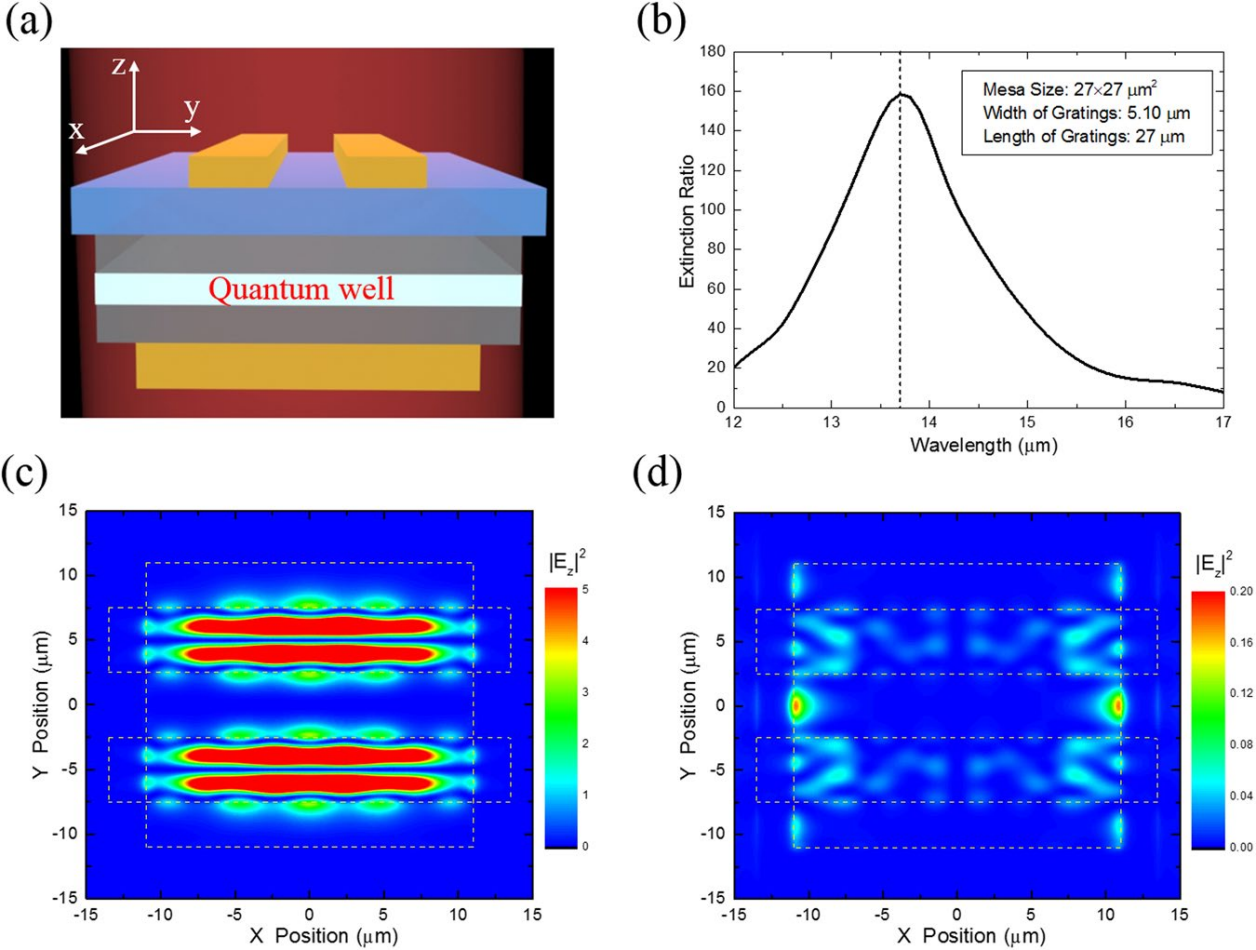
3D Simulation results of extinction ratio. (**a**) Schematic diagram of the 3D simulation. (**b**) 3D simulation results of extinction ratio. (**c**) Field map of 3D simulated ${|{{\rm{E}}}_{{\rm{z}}}|}^{2}$ in the quantum well (${\rm{\lambda }}={\rm{13.7}}\,{\rm{\mu }}{\rm{m}},\,{\rm{s}}={\rm{5.10}}\,{\rm{\mu }}{\rm{m}}$) with the TM polarized incident light. (**d**) Field map of 3D simulated ${|{{\rm{E}}}_{{\rm{z}}}|}^{2}$ in the quantum well (${\rm{\lambda }}={\rm{13.7}}\,{\rm{\mu }}{\rm{m}},\,{\rm{s}}={\rm{5.10}}\,{\rm{\mu }}{\rm{m}}$) with the TE polarized incident light.

We have demonstrated the high-polarization-discriminating PMC-QWIP super pixel with four channels, and the experimental extinction ratio of super pixel is above 100. What we are interested in is the origin and sustainability of the high ER of super pixel. We try to find the answer from the theoretical numerical simulation by COMSOL Multiphysics based on the finite element method. The 3D model of numerical simulation is shown in Fig. 5a, which has the same parameters as our real device. Using the same method with our previous work^24^ to set the material parameters^25^ of simulation, we carry out the numerical simulation by COMSOL Multiphysics.

Quantum well can only absorb the light of the electric field component along the growth direction of material (z direction in this paper), and many literatures^26,27^ show the quantum efficiency or photo-responsivity of QWIP is proportional to the $\overline{{|{{\rm{E}}}_{{\rm{z}}}|}^{2}}$ in the active region. The form of $\overline{{|{{\rm{E}}}_{{\rm{z}}}|}^{2}}$ is expressed as follows:

8 $$\overline{{|{{\rm{E}}}_{{\rm{z}}}(\lambda )|}^{2}}=\frac{1}{{\rm{V}}}{\int }_{{\rm{200nm}}}^{{\rm{207}}+{\rm{200nm}}}{\int }_{0}^{{\rm{27}}{\rm{\mu }}{\rm{m}}}{\int }_{0}^{{\rm{27}}{\rm{\mu }}{\rm{m}}}{|{{\rm{E}}}_{{\rm{z}}}({\rm{x}},{\rm{y}},{\rm{z}},{\rm{\lambda }})|}^{2}{\rm{dxdydz}}$$

where V is the volume of the active region (QW). The parameters of the structure have been shown in Fig. 1b. For the same device, the spectral response is approximately proportional to the $\overline{{|{{\rm{E}}}_{{\rm{z}}}|}^{2}}$ with the same intensity of incident light. Therefore, combined with the Equation (3), the simulated extinction ratio can be expressed as follows:

9 $${{\rm{ER}}}_{{\rm{simu}}}({\rm{\lambda }})=\frac{{{\rm{R}}}_{{\rm{TM}}}({\rm{\lambda }})}{{{\rm{R}}}_{{\rm{TE}}}({\rm{\lambda }})}=\frac{\overline{{|{{\rm{E}}}_{{\rm{z}},{\rm{TM}}}({\rm{\lambda }})|}^{2}}}{\overline{{|{{\rm{E}}}_{z,\mathrm{TE}}({\rm{\lambda }})|}^{2}}}$$

The 3D numerical simulation results of extinction ratio are shown in Fig. 5b. The peak of simulated extinction ratio is around 160 (DoLP = 0.988) at the wavelength of 13.7 μm. The experimental values of extinction ratio fit well with the results of 3D numerical simulation.

The numerical simulation results indicate that the PMC structure with one-dimensional (1D) gratings has a high mode selectivity. The TM polarized incident light is able to couple into the certain microcavity, and the TE polarized incident light can not couple into the microcavity. On one hand, the excellent mode selectivity of PMC structure determines that only the certain state of polarization can couple into the microcavity and form Fabry-Perot resonance. On the other hand, quantum well can only absorb the light of the electric field component along the growth direction of material according to the quantum selection rule of intersubband transition. That is to say, QWIPs just have responses to the certain polarized incident light that is perfectly selected by the designed PMC structure. This is the reason why the PMC-QWIP super pixel has such an excellent performance in polarization detection. Due to this unique advantage, the extinction ratio of PMC-QWIP super pixel is more than an order of magnitude larger than that of other monolithic integrated LWIR polarization detection super pixels.

### The sustainability of high extinction ratio of PMC-QWIP super pixel

If the length of 1D gratings is infinitely large, the extinction ratio should also be infinitely large in theory. However, things will change when the length of gratings is finite, especially when it comes to the pixel-level mesa. The size of the gratings is limited, therefore the decrease in length would lead to the rapid decrease of extinction ratio. Figure 5c,d show the field maps of the simulated distribution of ${|{{\rm{E}}}_{{\rm{z}}}|}^{2}$ in XOY plane with TM and TE polarized incident light respectively. It can be clearly seen from the field maps that there is a good coupling in the microcavity^28^ with the TM polarized incident light. It is the third-order localized surface plasmon (LSP) mode. The ${|{{\rm{E}}}_{{\rm{z}}}|}^{2}$ with the TM polarized incident light is mainly distributed in the microcavity around the center, and it is obviously weak near the edge of microcavity length. The situation is just the opposite when it comes to the TE polarized incident light. The ${|{{\rm{E}}}_{{\rm{z}}}|}^{2}$ with the TE polarized incident light is mainly distributed around the edge of microcavity length including the edge of both gratings and Au mirror, and the incident light is difficult to couple into the microcavity. It suggests that a small amount of TE polarized incident light leaks into the microcavity from the edge of length direction, which leads to a weak response of PMC-QWIP super pixel. Thus the extinction ratio is no longer infinitely large when the length of microcavity is limited. It could also be indicated that the gratings length of PMC-QWIP super pixel plays an important role in the sustainability of extinction ratio.

Actually, in practical application we are more concerned with the measured extinction ratio of super pixel. There are many factors that affect the measured extinction ratio, mainly including the following aspects:

1. The factors of fabrication such as the alignment precision of the metal gratings in photolithography, the roughness of the edge of metal gratings and so on;
2. The factors of experiments such as the noise in experiments (especially when ${{\rm{R}}}_{{\rm{\min }}}({\rm{\lambda }})$ is very small), the quality of polarizer, the pre-calibration of polarizer angle and so on;
3. The factors of microcavity parameters such as the size of metal gratings, the thickness of PMC, the size of metal mirror and so on.

## Conclusion

In this paper, we have developed the super pixel with its four subpixels’ gratings oriented at 0°, 45°, 90° and 135° respectively. This kind of super pixel is based on plasmonic microcavity quantum well infrared photodetectors for LWIR polarization imaging detection. Due to the double selection of both plasmonic microcavity and quantum well, the highest polarization ER measured with Fourier transform infrared spectrometer in experiment is 136, which is the highest reported value of LWIR polarization imaging detection super pixel. The excellent mode selectivity of plasmonic microcavity with 1D gratings is suggested by the results of three-dimensional (3D) theoretical simulation. The experimental responses of the super pixel with four polarization directions are in excellent agreement with the Malus’ Law. We accurately resolve the first three elements of the Stokes vector simultaneously at the pixel level. The QWIP-based plasmonic microcavity super pixel has shown its unique advantage in polarization detection, and it has a broad prospect and great potential to be developed into a new generation of high polarization discriminating LWIR-FPA.

## Methods

### Methods of fabrication

On a GaAs wafer, a 907 nm-thick dielectric layer was grown by molecular beam epitaxy (MBE). The order of growth was as follow: a 300 nm Al_0.5_Ga_0.5_As etch stop layer, a 200 nm n-doped GaAs bottom contact layer (doping with Si: ${\rm{n}}=2\times {10}^{17}$ cm^−3^), a 207 nm single quantum well layer (100 nm Al_0.15_Ga_0.85_As/7 nm n-doped GaAs/100 nm Al_0.15_Ga_0.85_As) and a 200 nm n-doped GaAs top contact layer (${\rm{n}}=2\times {10}^{17}$ cm^−3^). The mesa and common electrode were prepared by inductively coupled plasma etching^29^ (Oxford Plasmalab System 133) and wet chemical etching respectively, and the depth was controlled to reach the bottom contact layer. An AuGe (100 nm)/Ni (20 nm)/Au (300 nm) contact metal layer was deposited by electron-beam evaporation (ULVAC ei-5z), then after a lift-off process the top and bottom electrodes were formed simultaneously. The Ohmic contact was formed by rapid thermal annealing (AccuThermo AW 610). A Ti (50 nm)/Au (300 nm) reflection layer was deposited by electron-beam evaporation, then a 300 nm SiN_x_ passivation layer was deposited by plasma-enhanced chemical vapor deposition (PECVD). After a standard flip-chip bonding (Suss MicroTec FC150) with the fanout circuit using indium bumps and an underfill epoxy process, the chip was thinned to around 25 ${\rm{\mu }}m$ by a mechanical polishing process (Logitech 1PM51-1). A high selectivity etchant (mixture of citric acid (C_6_H_8_O_7_) and hydrogen peroxide (H_2_O_2_)) was used to remove the rest of the GaAs from the etch stop layer. The alignment markers were prepared by wet chemical etching, and the Ti (50 nm)/Au (150 nm) gratings were formed by electron-beam evaporation and a lift-off process. The PMC-QWIP device was finally fabricated. The 3D schematic diagram of the super pixel is shown in Fig. 1a.

### Methods of measurements

We use Fourier transform infrared spectrometer (FTIR) to measure the photocurrent spectra of the PMC-QWIP and the 45 degree polished facet device, and we obtain the photo-responsivity spectra and enhancement spectra with the blackbody responsivity. The polarization characteristics of the super pixel experiments are carried out by putting an angle-variable polarizer (ER > 600) between the dewar window and the FTIR and measuring the photocurrent spectra with different angles of polarized incident light. Due to an uncontrollable initial angle of the sample in dewar, we need to do a long-time angle calibration to ensure the accuracy of the angle everytime we test the polarization extinction ratio (ER). We define that 0 degree of the polarizer is when the direction of polarizer gratings is parallel to the direction of 0 degree gratings (G0) of the super pixel, and the value of angle on the polarizer increases clockwise (the reading becomes larger when we anticlockwise rotate the polarizer).
